# Supplementary material for: Predicting Hearing Loss in Testicular Cancer Patients after Cisplatin-Based Chemotherapy
Source: Cancers (Basel). 2023 Aug 1;15(15):3923. doi: 10.3390/cancers15153923 (PMC10417151; doi:10.3390/cancers15153923)
Supplement: Supplementary file 1 [file cancers-15-03923-s001.zip › cancers-2485666-supplementary.pdf]

Table of Contents

SUPPLEMENTARY MATERIAL **1**

Supplementary Table S1 .... **1**

Supplementary Figure S1 Step-by-step demonstration of genetic data quality control and information on patients where questionnaire information was missing. **2**

Supplementary Figure S2 Illustration of logistic regression model used in this study. **3**

Supplementary Figure S3 Misclassified patients and/or patients where genetic "pushed" final classification in the wrong direction. **4**

Supplementary Figure S4 ROC-AUC mean (30 random splits) performances for the random and real models. **5**

Supplementary Note S1 FACT/GOG-Ntx (Version 4). Hearing loss as part of ototoxicity. **6**

Supplementary Note S2 Genes obtained from database search. **7**

Supplementary Note S3 Model hyperparameters, encoding, and normalization **9**

SUPPLEMENTARY MATERIAL

Supplementary Table S1 Number of times each feature is selected in all 30 models. Each "Feature no. X" column theoretically sums up to 30, however, for illustration purposes, only eight features (best ROC-AUC performance) are shown in the table.

	**Feature no.1**	**Feature no.2**	**Feature no.3**	**Feature no.4**	**Feature no.5**	**Feature no.6**	**Feature no.7**	**Feature no.8**	**Total**
**Age at diagnosis**	30								30
**Treatment cycles**		30							30
***SOD2* rs4880**			3	4	2	2	4		15
***MGST3* rs9333378**			3		4	1	1		9
**Intergenic rs4389005**			3	1		1	1	1	7
***ABCA10* rs10491178**			1		1		1	3	6
***ABCA12* rs10498027**			1			2	1	1	5
***MCM8* rs3761873**						1	2	1	4

Step	Number of patients	Number of SNPs
Raw data	478	964,193
Prepared input	478	921,861
Low call rates	455	873,835
Gender check	455	873,835
Excess hetero- and homozygosity	454	873,835
Non CEU population	452	873,835
Relatedness	450	873,835
Population outliers	450	873,835
Non HWE and low MAF	450	611,129
Missing Questionnaire Information	401	611,129
Missing Label (NTX6)	393	611,129

Supplementary Figure S1 Step-by-step demonstration of genetic data quality control and information on patients where questionnaire information was missing. Single-nucleotide polymorphism (SNP) quality filtering included removal of duplicated SNPs and those with ambiguous genome position, strand, and alleles; call rate (<98%); extreme deviation from Hardy–Weinberg equilibrium (P value$<5 \times 10^{-6}$); and MAF (<1%). Quality controls applied on the patient samples were based on genotype (chromosome X homozygosity rate < 20% for females and > 80% for males) and phenotype sex discordance; extreme heterozygosity or homozygosity (±4 SD from sample's hetero-/homozygosity rate mean); outliers from the European descent using 1000 Genomes(49) as reference samples; cryptic relatedness (IBD>18.75%); and population outliers (±4 SD from cluster centroid mean). European outliers were detected by 1) doing principal component analysis (PCA) to find the center of the European reference samples, and 2) remove samples whose Euclidean distance from the center > 1.5 * maximum Euclidean distance of the European reference samples(50).

Patients with missing questionnaire information consisted of 45 patients who received more than one line of treatment and therefore were not relevant for the present study and were not invited for the questionnaire in 2014. These were still included for the purpose of quality control only.

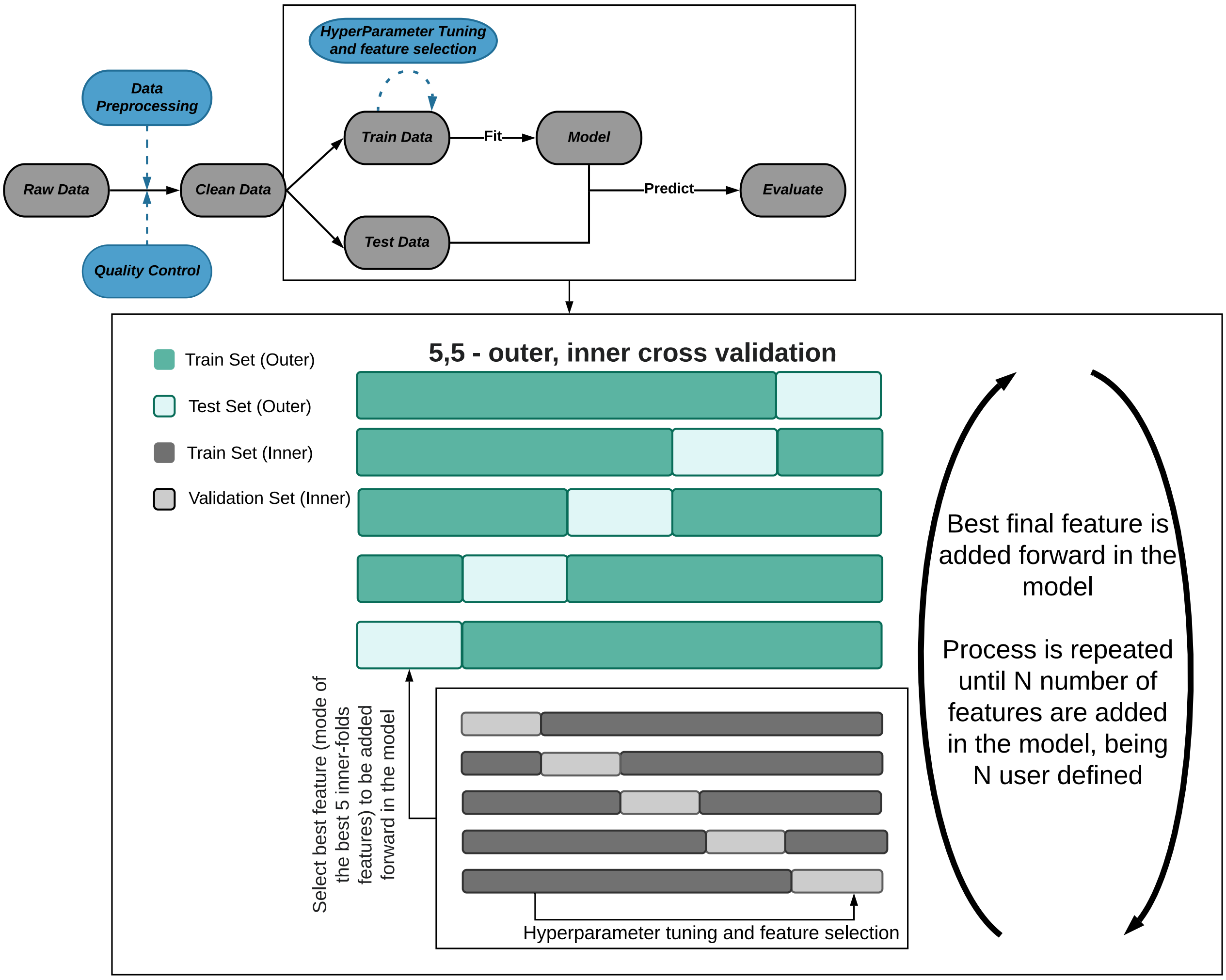

Supplementary Figure S2 Illustration of logistic regression model used in this study. Model was run at Computerome 2.0 (https://www.computerome.dk). The 30 random data splits were run in parallel to reduce running time, thus 32 nodes were used (30 allocated for each random split and 2 for other initializations). Each node contains 2 CPUs with 20 cores/CPU. 192 GB is the memory distributed through all cores.

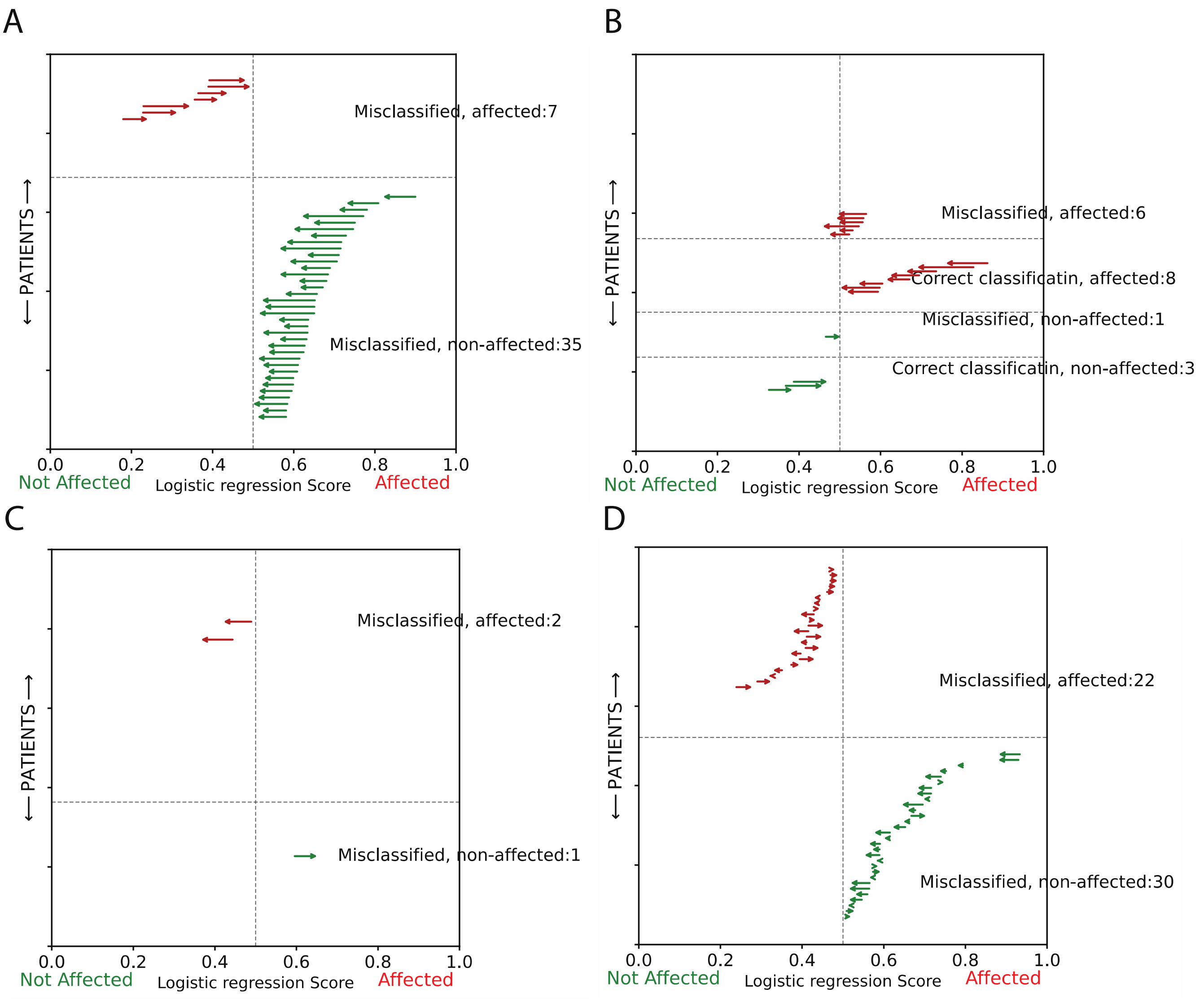

Supplementary Figure S3 Misclassified patients and/or patients where genetic "pushed" final classification in the wrong direction. Arrow starts at prediction score of model with only clinical data (model 1) and ends at prediction score of model with clinical and genetic data (model 2). **A:** Inclusion of genetic data helped but not enough to correctly classify these patients; **B:** Patients where genetic data "pushed" the classification in the wrong direction, even though some of them were correctly classified; **C, D:** Neither clinical nor genetic data helped on these patients classification (in **D,** score difference between model 2 and 1 was below 0.05). All other patients not represented here were correctly classified and genetic data "pushed" the classification in the right direction (or if in the wrong direction, score difference between model 2 and model 1 was below 0.05).

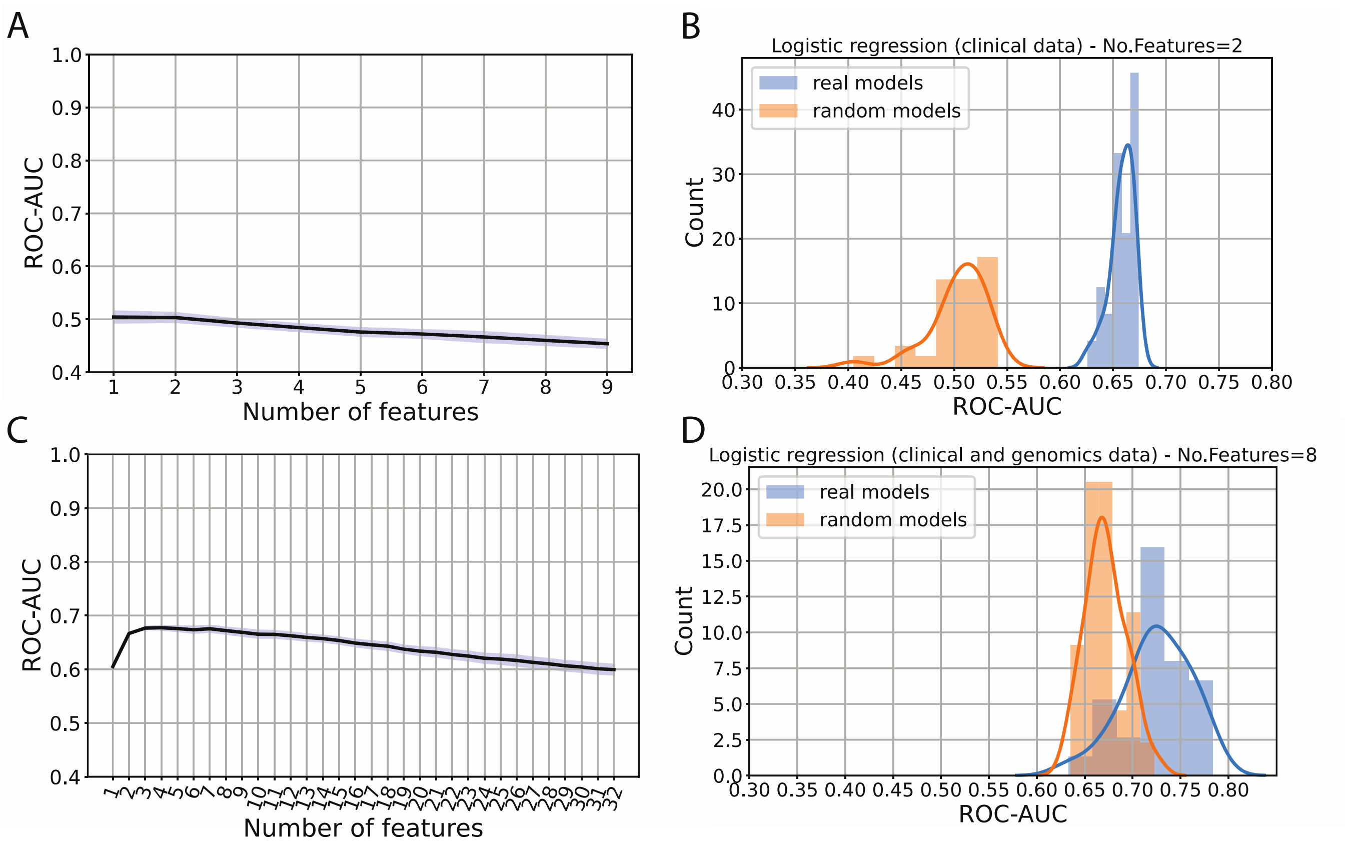

Supplementary Figure S2 ROC-AUC mean (30 random splits) performances for the random and real models**.** A: Model with permuted clinical data with forward feature selection up until nine features. Shaded blue area indicates 95% CI. B: Comparison between real model (mean ROC-AUC 0.66 (95% CI, 0.65-0.66, blue histogram) and permuted models (mean ROC-AUC 0.50 (95% CI, 0.49-0.51, orange histogram); C: Model with clinical and randomly selected markers with forward feature selection up until 32 features. Shaded areas indicate 95% CI. D: Comparison between real model (mean ROC-AUC of 0.73 (95% CI, 0.71-0.74) and random models (mean ROC-AUC was 0.67 (95% CI, 0.66-0.68). In B and D, count (y-axis) sums up to 150 as the model consists of 5 outer folds and 30 data splits were done (5x30).

ROC-AUC = area under the receiver operating characteristic curve; No.= number; CI = confidence interval.

Supplementary Note S1 FACT/GOG-Ntx (Version 4). Hearing loss as part of ototoxicity.

	Not at all	A little bit	Somewhat	Quite a bit	Very much
**FACT/GOG-Ntx6:** I have trouble hearing	0	1	2	3	4
**FACT/GOG-Ntx7:** I get a ringing or buzzing in my ears	0	1	2	3	4

Supplementary Note S2 Genes obtained from database search.

Resistance Pathway (KEGG Pathways*) (with aliases):
GST, gst, PIK3CA, APAF1, BAD, BAX, BCL2, CASP3, REV3L, POLZ, FADD, PIK3R1, TOP2, POLH, ERK, MAPK1, TNFSF6, FASL, CD178, TNFRSF6, FAS, CD95, CASP8, CASP9, MAP3K5, ASK1, TP53, P53, AKT, BCL2L1, bcl-xL, XIAP, BIRC4, BID, ATM, TEL1, ERBB2, HER2, CD340, ABCC2, PDPK1, CDKN2A, P16, INK4A, CDKN1A, P21, CIP1, MDM2, BIRC5, MLH1, MSH2, MSH3, MSH6, CYC, PMAIP1, NOXA, BBC3, PUMA, BRCA1, XPA, ERCC1, BAK, BAK1, SLC31A1, CTR1, BIRC2, copA, ctpA, ATP7, GSTP, BIRC3, MAPK3, PIK3R3, PIK3R2, PIK3CB, PIK3CD
***KEGG entry name: "Platinum drug resistance"**

Detoxification Pathway (BioCyc Pathway)
GSTZ1, GSTA1, GSTA2, ABCC1, GGT1, GGT5, CCBL1, CCBL2, NAT8
***Name of pathway in BioCyc: "glutathione-mediated detoxification I"**

Glutathione Transferases (Uniprot*) (with aliases)
PTGES, MGST1L1, MPGES1, PGES, PIG12, MGST3, MGST2, GST2, MGST1, GST12, MGST, LANCL1, GPR69A, HPGDS, GSTS, PGDS, PTGDS2, GSTZ1, MAAI, GSTT4, GSTTP1, GSTT2B, GSTT2, GSTT2, GSTT1, GSTP1, FAEES3, GST3, GSTO2, GSTO1, GSTTLP28, GSTM5, GSTM4, GSTM3, GST5, GSTM2, GST4, GSTM1, GST1, GSTK1, HDCMD47P, GSTCD, GSTA5, GSTA4, GSTA3, GSTA2, GST2, GSTA1
***search string: name:glutathione name:transferase AND reviewed:yes AND organism:"Homo sapiens (Human) [9606]"**

Cytochrome P450 enzymes (Uniprot*) (with aliases)
CYP2C9, CYP2C10, CYP21A2, CYP21, CYP21B, CYP3A4, CYP3A3, CYP2C8, CYP27B1, CYP1ALPHA, CYP27B, CYP26B1, CYP26A2, P450RAI2, CYP1A1, CYP11B2, CYP4A11, CYP4A2, CYP51A1, CYP51, CYP26A1, CYP26, P450RAI1, CYP2C19, CYP17A1, CYP17, S17AH, CYP1A2, CYP11B1, S11BH, CYP2A6, CYP2A3, CYP2C18, CYP27A1, CYP27, CYP3A5, CYP2B6, CYP2D6, CYP2DL1, CYP11A1, CYP11A, CYP4F2, CYP2E1, CYP2E, CYP4B1, CYP3A7, CYP4F3, LTB4H, CYP24A1, CYP24, CYP19A1, ARO1, CYAR, CYP19, CYP2A7, CYP7A1, CYP7, CYP4A22, CYP2F1, CYP4F12, UNQ568, PRO1129, CYP39A1, CYP7B1, CYP2A13, CYP26C1, CYP46A1, CYP46, CYP2U1, POR, CYPOR, CYP4F11, CYP4F22, CYP4V2, CYP2W1, CYP2S1, UNQ891, PRO1906, CYP2J2, CYP2R1, CYP4F8, CYP1B1, CYP3A43, CYP2D7, CYP27C1, TBXAS1, CYP5, CYP5A1, CYP8B1, CYP12, CYP4X1, UNQ1929, PRO4404, CYP4Z1, UNQ3060, PRO9882, CYP2G1P, CYP2GP1, CYP20A1, UNQ667, PRO1301, CYP4Z2P, CYP4F30P, C2orf14
***search string: name:cytochrome name:p450 AND reviewed:yes AND organism:"Homo sapiens (Human) [9606]"**

ATP binding cassette (Uniprot*) (with aliases)
TAP2, ABCB3, PSF2, RING11, Y1, TAP1, ABCB2, PSF1, RING4, Y3, CFTR, ABCC7, ABCG8, ABCG5, ABCG4, WHITE2, ABCG2, ABCP, BCRP, BCRP1, MXR, ABCG1, ABC8, WHT1, ABCD4, PXMP1L, ABCD3, PMP70, PXMP1, ABCD2, ALD1, ALDL1, ALDR, ALDRP, ABCD1, ALD, ABCC9, SUR2, ABCC8, HRINS, SUR, SUR1, ABCC6, ARA, MRP6, ABCC5, MRP5, ABCC4, MRP4, ABCC3, CMOAT2, MLP2, MRP3, ABCC2, CMOAT, CMOAT1, CMRP, MRP2, ABCC12, MRP9, ABCC11, MRP8, ABCC10, MRP7, SIMRP7, ABCC1, MRP, MRP1, ABCB9, KIAA1520, ABCB8, MABC1, MITOSUR, ABCB7, ABC7, ABCB6, MTABC3, PRP, UMAT, ABCB5, ABCB4, MDR3, PGY3, ABCB11, BSEP, ABCB10, ABCB1, MDR1, PGY1, ABCA9, ABCA8, KIAA0822, ABCA7, ABCA6, ABCA5, KIAA1888, ABCA4, ABCR, ABCA3, ABC3, ABCA2, ABC2, KIAA1062, ABCA13, ABCA12, ABC12, ABCA10, ABCA1, ABC1, CERP
***search string: name:atp name:binding name:cassette AND reviewed:yes AND organism:"Homo sapiens (Human) [9606]"**

Cisplatin (Uniprot*) (with aliases)
ATP11B, ATPIF, ATPIR, KIAA0956, LRRC8D, LRRC5, UNQ213, PRO239, SSRP1, FACT80, RAD23B, SIVA1, SIVA, PRIMPOL, CCDC111, LRRC8A, KIAA1437, LRRC8, SWELL1, UNQ221, PRO247, MCM8, C20orf154, ABCC2, CMOAT, CMOAT1, CMRP, MRP2, POLH, RAD30, RAD30A, XPV, SLC22A2, OCT2, SRSF2, SFRS2, DCLRE1A, KIAA0086, SNM1, SNM1A, FAM168A, KIAA0280, TCRP1, RDM1, RAD52B, XPC, XPCC, YBX1, NSEP1, YB1, NOX3, MOX2, ADIRF, AFRO, APM2, C10orf116, DNAJC15, DNAJD1, GIG22, HSD18, TMEM205, UNQ501, PRO1018, CLPTM1L, CRR9
***search string: (annotation:(type:function cisplatin) OR annotation:(type:"activity regulation" cisplatin) OR annotation:(type:disease cisplatin) OR annotation:(type:pharmaceutical cisplatin) OR annotation:(type:mutagen cisplatin)) AND reviewed:yes AND organism:"Homo sapiens (Human) [9606]"**

Cisplatin (Drugbank*)
MPG, A2M, TF, ATOX1, MPO, XDH, CYP4A11, PTGS2, nat, CYP2C9, CYP2B6, BCHE, GSTT1, MT1A, MT2A, SOD1, GSTP1, NQO1, GSTM1, ALB, ABCC3, ABCC5, ABCC2, SLC22A2, SLC31A1, SLC31A2, ABCC6, ABCB1, ATP7B, ATP7A, ABCG2
***Name of drug in Drugbank: "cisplatin"**

Sensorineural hearing loss (Uniprot*) (with aliases)

ABHD12, ACS4, ACSL4, ACTG, ACTG1, ADMLX, AFG2, AIE75, AIGF, ALR, AMMECR1, AMMECR2, ANOS1, AP19, AP1S1, APNH1, ARSG, ATP1A3, ATP6B1, ATP6B2, ATP6N1B, ATP6N2, ATP6V0A4, ATP6V1B1, ATP6V1B2, AXOR12, BCS1, BCS1L, BFGFR, BHLHE32, BM28, BOM, BRWD2, BV8, C14orf10, C19orf64, C1orf7, C20orf22, C20orf54, C21orf29, C6orf125, C6orf29, C6orf32, C9orf75, C9orf81, CCNL1, CD164, CDC14A, CDCL1, CDH23, CEACAM16, CEAL2, CEK, CEP2, CEP250, CEP78, CGI-47, CHD7, CIAS1, CIB2, CLAPS1, CLDN14, CLLD7, CNAP1, COI, COL11A1, COL11A2, COL2A1, COL4A6, COL9A2, COLL6, COMT2, COXI, CRYM, CTL4, DCDC2, DFNA5, DFNB31, DFNB36, DIABLO, DIAP1, DIAP3, DIAPH1, DIAPH3, DIFF48, DLX5, DMXL2, DUSP6, E4.5, ECHOS1, EDG5, EIF3F, EIF3S5, ELMOD3, ENT3, EPS8L2, EPS8R2, ESPN, EXOSC2, EYA4, FACL4, FAM65B, FER1L2, FEZ, FEZF1, FGF17, FGF8, FGFBR, FGFR1, FGFR3, FKHL7, FLG, FLRT3, FLT2, FOXC1, FP17425, FREAC3, G5PR, GAS3, GFER, GIPC3, GJB2, GJB6, GMPPB, GNRH, GNRH1, GNRHR, GPR54, GPR73L1, GRAP, GRH, GRHL2, GRHR, GSDME, HBGFR, HCCS4, HERV1, HGF, HOMER2, HPO, HPTA, HRIHFB2122, HS6ST, HS6ST1, HXB, IARS2, ICERE1, IL17RD, IL17RLM, ILDR1, IRX2A, IRX5, IRXB2, JTK4, KAL, KAL1, KALIG1, KCNE1L, KCNE5, KCNJ10, KCNQ4, KIAA0030, KIAA0386, KIAA0389, KIAA0567, KIAA0772, KIAA0856, KIAA1001, KIAA1154, KIAA1171, KIAA1351, KIAA1416, KIAA1469, KIAA1526, KIAA1662, KIAA1774, KIAA1812, KIAA1897, KIAA2034, KIP2, KISS1, KISS1R, KRML, LACS4, LHFPL5, LHRH, LP2654, LRP2, LRTOMT, MAFB, MAP3K20, MARS2, MARVELD2, MCM2, MET, MITF, MKP3, MKS3, MLTK, MNF1, MT-CO1, MTCO1, MYH14, MYH9, MYO15, MYO15A, MYO1F, MYO6, MYO7A, NALP3, NELF, NG22, NHE1, NK3R, NKNB, NLRP3, NRSF, NSMF, NTRKR1, OPA1, ORP2, OSBPL2, OTOA, OTOF, P2RX2, P2X2, PAF1, PAF3, PCDH15, PCNA, PDS, PEX1, PEX10, PEX12, PEX13, PEX2, PEX26, PEX5, PEX6, PI6, PKR2, PL48, PMP22, PMP3, PMP35, PP13181, PP4068, PP5098, PP7517, PPP2R3C, PRES, PRO1155, PRO1380, PRO1571, PRO1777, PRO1865, PRO187, PRO20026, PRO382, PRO4340, PRO874, PROK2, PROKR2, PRPS1, PTI, PTPRQ, PUS7, PXAAA1, PXMP3, PXR1, PYPAF1, PYST1, RAB40AL, RBED1, RBM29, RCBTB1, RDX, REST, RFT2, RFVT3, RIPOR2, RLGP, RNF69, RNF72, ROR1, RRP4, RU2, S1PR2, SANS, SEF, SEMA3A, SEMAD, SERPINB6, SLC17A8, SLC26A4, SLC26A5, SLC29A3, SLC44A4, SLC52A3, SLC9A1, SLITRK6, SMAC, SPAF, SPATA5, SPRY4, STRC, TAC3, TAC3R, TACR3, TADG12, TARA, TBC1D24, TBL1, TBL1Y, TECTA, TFCP2L3, THBP, TMC1, TMEM132E, TMEM67, TMHS, TMIE, TMPRSS3, TNC, TOMT, TPPT1, TPRN, TRIC, TRIOBP, TRNT1, TSPEAR, TUBB2C, TUBB4B, UNQ161, UNQ1894, UNQ323, UNQ441, UNQ585, UNQ6115, UNQ717, UNQ777, UNQ839, UNQ856, UQCC2, USH1B, USH1C, USH1F, USH1G, USH2A, VATB, VGLUT3, VPP3, VPP3, WBP2, WDR11, WDR15, WFS1, WHRN, XBR, XPNPEP3, ZAK, ZNF312B

***search string: reviewed:yes AND organism:”Homo sapiens (Human) [9606]” AND (annotation:(type:disease ”sensorineural hearing loss”) OR annotation:(type:”disruption phenotype” ”sensorineural hearing loss”) OR annotation:(type:mutagen ”sensorineural hearing loss”) OR annotation:(type:function ”sensorineural hearing loss”) OR annotation:(type:pathway ”sensorineural hearing loss”))**

Ototoxicity (Uniprot*) (with aliases)

TRMU, MTU1, TRMT1, MYO7A USH1B

***search string: reviewed:yes AND organism:”Homo sapiens (Human) [9606]” AND (annotation:(type:disease *Search string: ”ototoxicity”) OR annotation:(type:”disruption phenotype” ”ototoxicity”) OR annotation:(type:mutagen ”ototoxicity”) OR annotation:(type:function ”ototoxicity”) OR annotation:(type:pathway ”ototoxicity”))**

Supplementary Note S3 Model hyperparameters, encoding, and normalization

On the logistic regression model, hyperparameter optimization was done for the inverse of regularization strength. Values between $1x10^{-4}$ and $1x10^{4}$ were tried (20 values in total spaced evenly on a log scale). Smaller values specify stronger regula
rization.

Ordinal categorical variables were encoded as one-column vectors, i.e. ({1,2,3,4}) for treatment cycles 3, 4, 5 or more, and high dose (double dose of chemotherapy, unspecified number of cycles) BEP cycles, respectively; ({1,2,3}) for prognosis good, intermediate, and poor, respectively; and ({0,1,2}) for smoking habits never, former, and current, respectively. The nominal variable histology was encoded in one column, ({1,2}), for non-seminoma, or seminoma, respectively. Continuous variables were represented in absolute values (age at diagnosis, body mass index, glomerular filtration rate before treatment, cumulative cisplatin dose per square meter of BSA, and alcohol consumption in number of units per week. Single-nucleotide polymorphism data was encoded as one column vector with counts of minor alleles ({0,1,2}).

Features were scaled down to values between 0 and 1 using Sklearns MinMaxScaler. Rescaling of features was done separately in the training and test set to avoid leakage of the test set information.
